# An Expert, Multidisciplinary Perspective on Best Practices in Biomarker Testing in Intrahepatic Cholangiocarcinoma

**DOI:** 10.1093/oncolo/oyac139

**Published:** 2022-08-04

**Authors:** David C Madoff, Nadine Abi-Jaoudeh, David Braxton, Lipika Goyal, Dhanpat Jain, Bruno C Odisio, Riad Salem, Mark Schattner, Rahul Sheth, Daneng Li

**Affiliations:** Yale University School of Medicine, New Haven, CT, USA; University of California Irvine, Orange, CA, USA; Hoag Memorial Hospital Presbyterian, Newport Beach, CA, USA; Massachusetts General Hospital, Boston, MA, USA; Department of Pathology, Yale University School of Medicine, New Haven, CT, USA; The University of Texas MD Anderson Cancer Center, Houston, TX, USA; Northwestern University, Chicago, IL, USA; Memorial Sloan Kettering Cancer Center, New York, NY, USA; The University of Texas MD Anderson Cancer Center, Houston, TX, USA; City of Hope, Duarte, CA, USA

**Keywords:** intrahepatic cholangiocarcinoma, biomarker testing, interventional oncology, pathology, best practices, multidisciplinary

## Abstract

Intrahepatic cholangiocarcinoma (iCCA) is a rare and aggressive malignancy that arises from the intrahepatic biliary tree and is associated with a poor prognosis. Until recently, the treatment landscape of advanced/metastatic iCCA has been limited primarily to chemotherapy. In recent years, the advent of biomarker testing has identified actionable genetic alterations in 40%-50% of patients with iCCA, heralding an era of precision medicine for these patients. Biomarker testing using next-generation sequencing (NGS) has since become increasingly relevant in iCCA; however, several challenges and gaps in standard image-guided liver biopsy and processing have been identified. These include variability in tissue acquisition relating to the imaging modality used for biopsy guidance, the biopsy method used, number of passes, needle choice, specimen preparation methods, the desmoplastic nature of the tumor, as well as the lack of communication among the multidisciplinary team. Recognizing these challenges and the lack of evidence-based guidelines for biomarker testing in iCCA, a multidisciplinary team of experts including interventional oncologists, a gastroenterologist, medical oncologists, and pathologists suggest best practices for optimizing tissue collection and biomarker testing in iCCA.

## Teaser

There are challenges associated with biomarker testing in patients with cancer, including rare cancers such as intrahepatic cholangiocarcinoma. This article outlines best practices and recommendations from a multidisciplinary team of experts to assist physicians involved in the care of patients with intrahepatic cholangiocarcinoma.

**Implications for Practice**

Studies indicate that approximately 50% of patients with intrahepatic cholangiocarcinoma (iCCA) may have therapeutically targetable genetic alterations. Consequently, testing has become paramount for optimal care of patients with iCCA. There are current challenges associated with biomarker testing in patients with cancer, including rare cancers such as iCCA. Thus, it is expected that the best practices and recommendations from a multidisciplinary team of experts that are outlined in this article will greatly benefit academic and community physicians involved in the care of patients with iCCA in practicing precision medicine and improving patient care.

## Introduction, Materials, and Methods

A systematic review was not conducted for this article. A content outline was created by the authors and citations that best supported the content were included; the authors determined the value of each publication selected for inclusion based on their clinical experience without a formal level of evidence assessment. Unpublished material was not included. To avoid confusion and to be consistent with the adoption of consensus terms for testing in precision medicine, the term biomarker testing will be used hereafter when referring to testing that is performed to identify genetic alterations.^1^ While liquid biopsies are emerging as a significant tool for biomarker testing, a description of the technique and utilization of liquid biopsies for patients with intrahepatic cholangiocarcinoma (iCCA) is beyond the scope of the article.

CCA comprises a group of heterogeneous biliary tract malignancies associated with a poor prognosis due to their late-stage presentation, aggressive nature, and limited therapeutic options; 5-year survival rates for CCA are low (7%-20%).^2,3^ Based on their anatomical site of origin, CCAs are classified as intrahepatic (iCCA), perihilar (pCCA) and distal (dCCA).^2^ This review is focused on iCCA, a subset that arises from the intrahepatic biliary tree distal to the second-order bile ducts.^2,4,5^ Current evidence indicates that the incidence of iCCA has been steadily increasing; in the US, the incidence of iCCA increased from 0.44 cases per 100,000 in 1973 to 1.18 cases per 100,000 in 2012.^6-8^

The treatment landscape of advanced/metastatic iCCA largely remains palliative and has been limited primarily to chemotherapy. However, the past decade has witnessed the advent of biomarker testing and precision medicine that enables tailoring of targeted therapies to improve patient outcomes. While this has made biomarker testing using next-generation sequencing (NGS) increasingly relevant, recent studies have shown there are challenges with tissue acquisition for biomarker testing in patients with hepatobiliary cancers.^9-11^ Moreover, standard image-guided liver biopsy and processing guidelines for biomarker testing do not exist, creating challenges and gaps for the providers and their patients. To address these barriers, a multidisciplinary panel of healthcare providers who are key in acquiring tissue, coordinating NGS testing, and interpreting results—interventional oncologists (IO), a gastroenterologist (GI), medical oncologists (MO), and pathologists—were convened to discuss best practices in optimizing tissue collection for biomarker characterization in iCCA. The current review summarizes these discussions and expert perspectives.

## Current and Emerging Treatment Landscape and Rationale for Biomarker Testing in iCCA

The ABC-02 trial established gemcitabine-cisplatin as standard of care for the first-line treatment of patients with advanced biliary tract cancers (BTC) based on improved overall survival (OS, 11.7 vs 8.1 months; *P* < .001) and progression-free survival (PFS, 8.0 vs 5.0 months, *P* < .001) compared with gemcitabine alone.^12^ In the second-line setting, the ABC-06 trial recently demonstrated OS survival benefit (6.2 vs 5.3 months, *P* = .031) with modified FOLFOX plus Active Symptom Control (ASC) vs ASC alone.^13^ Despite these results, an unmet need persists for development of novel therapeutic options to improve outcomes for patients with iCCA.

Approximately 40% to 50% of patients with iCCA are expected to have potentially actionable targets, including genetic aberrations in isocitrate dehydrogenase-1 (*IDH1*; 10-20%), fibroblast growth factor receptor 2 (*FGFR2*; 10-16%), *BRAF* mutations (<5%), *NTRK* fusions (<5%), and MSI-H/dMMR, TMB >10 mutations/megabase (<5%)^14^ (**Table 1**). Targeted therapies with selective inhibitors have demonstrated clinical efficacy and safety in iCCA. Treatment with FGFR inhibitors results in response rates of 23%-42%, disease control rates ~80%, and mPFS of 7-9 months^15-17^ in previously treated patients with advanced iCCA harboring *FGFR2* fusions/rearrangements. Based on efficacy and safety results of multi-center, open-label clinical trials, pemigatinib and infigratinib have gained accelerated approval by the US Food & Drug Administration (FDA) for the treatment of patients with previously treated, unresectable locally advanced or metastatic CCA with an *FGFR2* fusion or other rearrangement.^18,19^ Other FGFR inhibitors are in development and are further being tested in both the second- and first-line settings.^20^ In addition, based on demonstration of significant PFS improvement in the placebo-controlled phase III ClariDHy trial, the FDA recently approved the IDH1 inhibitor ivosidenib for adult patients with previously treated, locally advanced or metastatic CCA with an *IDH1* mutation.^21,22^ Other targeted therapies that have shown encouraging activity in BTC include dabrafenib plus trametinib for *BRAFV600E*-mutated BTC^23^ and pembrolizumab for tumors with deficient mismatch repair (dMMR) proteins/high microsatellite instability (MSI-H).^24,25^ The National Comprehensive Cancer Network (NCCN) classifies their recommendations for these therapies as Category 2A.^26^

**Table 1.** Actionable genetic aberrations in iCCA.

Gene	Prevalence*
*IDH1* mutations	13%-20%
FGFR2 fusions/rearrangements	10%-16%
*BRAF* mutations	<5%
NTRK fusions	<5%
MSI-high/dMMR, TMB >10 mutations/megabase	<5%

*The percentages provided are approximations.

Source: Cho et al.^14^

Abbreviations: dMMR, deficient mismatch repair; FGFR, fibroblast growth factor receptor; IDH1, isocitrate dehydrogenase-1; MSI, microsatellite instability; TMB, tumor mutational burden.

Recognizing the emerging role of precision medicine for the management of iCCA, expert guidelines and working groups, including the NCCN and European Society for Medical Oncology (ESMO), recommend biomarker testing for patients with unresectable and metastatic iCCA.^26,27^

## Diagnosis of iCCA with Tissue Samples

The identification of actionable targets and use of targeted therapies requires a diagnosis of iCCA. The diagnosis of iCCA requires a high level of suspicion in the appropriate clinical setting and a panel of confirmatory clinical, histologic, and imaging data.^28^ However, accurate diagnosis of iCCA is notoriously challenging, largely because of the lack of clear diagnostic imaging criteria and the absence of specific serum tumor and immunohistochemical markers.^2,3,28,29^

The major challenge in iCCA diagnosis is distinguishing it clinically from other hepatic lesions such as hepatocellular carcinoma (HCC) and metastatic lesions. Imaging data are critical for the diagnosis of iCCA, particularly for differentiating it from HCC; however, there are currently no accepted imaging criteria that provide a definitive diagnosis.^2,28^ On dynamic CT or MRI, iCCA shows venous phase contrast enhancement while HCC shows arterial phase enhancement plus delayed venous phase washout, which can aid in differentiating between iCCA and HCC.^28^

Circulating tumor markers are of moderate value, CA19-9 may be helpful but the sensitivity and specificity for iCCA is only 62% and 63%, respectively.^28^ Histologically, iCCA is nonspecific, showing adenocarcinoma with prominent stromal desmoplasia; some forms are somewhat characteristic while others can mimic a wide range of other tumors.^28,30^ The immunohistochemical profile of these tumors is also not specific, significantly overlapping with other carcinomas.^28,30^

Recent evidence suggests that albumin, one of the best characterized markers of hepatic progenitor cells, may be a good biomarker of iCCA but does not distinguish from other primary intrahepatic malignancies such as HCCs.^31^ Novel characteristic histologic features of iCCA may also be useful, including the recently described cholangiolar pattern that is comprised of well-formed ducts with angular profiles that mimic antler horns.^31^ Lastly, presence of alterations in *FGFR2*, *IDH1*, and *BAP-1* oncogenes on biomarker testing, especially in some combination, can strongly support iCCA diagnosis.^32^

## Challenges in Biomarker Testing in BTC

Acquiring sufficient tissue for biomarker testing is a challenge in biliary tract tumors, including iCCA.^9-11,29^ This can be partly attributed to the unique location of the tumors and the high stromal and desmoplastic nature of CCA that can lead to low tumor cell content in acquired biopsies. In 123 tissue samples analyzed from patients with advanced BTC (iCCA: 68.2%), 26.8% of samples failed NGS analysis, predominantly due to insufficient archival tumor content (<20%) and low DNA extraction for analysis.^9^ Moreover, quality control for RNA fusions failed in 12 of 34 (35.3%) samples. The authors attributed the lower tumor content (<20%) to difficulty in collecting optimal biopsies for patients with BTC and suggested that it may be improved by involving a pathologist for immediate assessment during the biopsy procedure. Similarly, the MOSCATO-1 trial included 43 patients with advanced BTC (67% being iCCA) and also demonstrated that biomarker testing was unsuccessful in 21% of patients.^10^

## Challenges of Tissue Collection and Processing

Barriers to optimized biopsies and tissue processing are multifactorial. Challenges associated with imaging modality, tissue acquisition method, sampling bias, tissue yield, specimen handling, and safety risks are discussed below.

### Imaging Modality

Liver biopsies are primarily performed under ultrasound (US) or computed tomography (CT) guidance. The preferred imaging modality used is based on parameters of the lesion (size, location, accessibility, and visualization), management of potential complications, equipment availability, and cost.^33^ While guidelines recommend US guidance to obtain liver biopsies, some lesions may require CT for optimal visualization.^33-35^

### Tissue Acquisition Method

There is no standardized biopsy technique for biomarker testing; both core needle biopsy (CNB) and fine needle aspiration (FNA) are used in clinical practice.^33,36,37^ Core needle biopsy provides a larger tissue yield and preserves tissue architecture yet has longer tissue fixation and processing time and is harder to acquire from small lesions.^38^ Fine needle aspiration has a more rapid turnaround time but does not provide morphological details; when used alone, it may not acquire sufficient tumor cellularity for biomarker testing.^38,39^

### Sampling Bias

Desmoplastic stroma and necrosis are complicating factors for iCCA biopsies and are associated with low tumor cell content, often reducing optimal yields for biomarker testing.^40,41^ In patients with refractory cancer with failed samples (n=61; failed samples had tumor cell content <30%), the presence of necrosis and fibrosis were both associated with biopsy failures (38% and 16%, respectively).^42^ In addition, mucin in stroma of liver metastases may contribute to poor tissue sampling.^39^

### Tissue Yield and Handling

Currently, the NGS-based biomarker testing platform defines the necessary cell fraction, tumor surface area, and amount of nucleic acid yield, along with directions regarding preanalytical tissue handling and preservation factors.^43,44^ Despite attempts to standardize tissue acquisition and processing, biomarker testing remains a challenge for patients with BTC. In a real-world study of >30 000 solid tumor samples that underwent a polymerase chain reaction (PCR)-based comprehensive genomic profiling (CGP) test, only 29.9% of biliary samples (*n* = 633) had tumor surface area >25 mm^2^ and 19.3% had tumor cell content <20%,^45^ both of which are under the standard thresholds for most NGS platforms.

### Safety Risks

Based on current evidence, the risk of a major complication is low with percutaneous liver biopsies, but may include bleeding, organ perforation, sepsis, needle track seeding, and death.^33,35,39^ Bleeding has been reported in up to 10% of cases, with major bleeding occurring in <2%.^35^ Moreover, needle track seeding rates of 0-4% have been reported, mostly after percutaneous biopsy of HCC.^33^ Risks of liver biopsy vary based on many factors, including number of passes.^46^ A recent study showed 3 or more passes compared to 1 pass significantly increased the risk of complications and morbidity from percutaneous liver biopsies (OR: 2.97; *P* = .0005).^47^

## Guiding Principles in Optimizing Tissue Collection and Processing

Several practices to improve tissue acquisition for biomarker testing in patients with iCCA are outlined below.

### Tissue Acquisition Methods

Current evidence supports the combined use of FNA and CNB to increase tumor cell content.^39,40,48,49^ Of relevance to iCCA, FNA favors extraction of loosely cohesive epithelial tumor cells while leaving behind dense connective tissue such as the tumor-associated stroma.^39,40^ On the other hand, CNB samples the entire target area including stromal and non-tumor components that are often associated with a lower concentration of tumor cells.^40^ In fact, to enhance biomarker testing success rates, recent clinical trials including BATTLE-2 and NCI-MATCH incorporated FNA with CNB.^39,50^ While the combined use of FNA and CNB for biomarker testing has been adopted by select clinical trials, this practice has not been standardized, suggesting the need for further research in multiple tumor types, including iCCA.

### Choice of Needle Gauge and Number of Passes

The choice of biopsy needles and number of passes vary depending on the risk-benefit and tissue requirements.^35,39,48,51^ While acquisition of multiple cores increases the likelihood of evaluable tissue, there are no established guidelines for the number of core specimens that need to be collected; 3-5 cores are typically collected for CNB and 2-3 for FNA.^39,48,52^ Jamshidi and colleagues showed that the total nucleic acid yield was 4.8-5.7 times greater when using an 18G needle versus a 20G needle and 2.4-2.8 times greater when using a double pass versus a single pass.^51^These results highlight the relative importance of needle gauge versus the number of passes effect on nucleic acid content.^51^

### Sample Bias, Tissue Processing and Handling: The Pathologist’s Perspective

Current evidence indicates that rapid on-site evaluation (ROSE) by cytologic evaluation of the FNA biopsy smears and/or touch preparations from core samples may improve tissue yield and ensure specimen adequacy.^39,53,54^ In fact, a retrospective analysis of 40 clinical research biopsies obtained at Duke University reported a success rate of 86% when real-time immediate cytopathologic assessment via telepathology was performed versus 65% when not performed.^39^ While ROSE may improve multidisciplinary communication and tissue processing, it may not be widely available due to cost, time, personnel, and resources.^55^

To ensure tissue is not exhausted prior to biomarker testing, it is essential to split each core into 2 specimens, one for diagnosis and one for biomarker testing.^39^ This process has been adopted in clinical trials, including BATTLE-2, where 2 CNB samples were used for histology quality control while the other 2 CNB samples were used for NGS biomarker testing.^50^ Additionally, once samples are preserved, macro/microdissection is also important for tumor enrichment, which is particularly relevant in desmoplastic tumor types such as iCCA.^39,44,55^ Microdissection was practiced in the NCI-MATCH trial and rendered otherwise unevaluable specimens evaluable for biomarker testing.^39^

### Best Practices in Communication Among the Multidisciplinary Team

A key element for optimizing biopsy outcomes is developing a multidisciplinary team-based approach that facilitates collaboration among interventional oncologists, medical oncologists, surgical oncologists, gastroenterologists, and pathologists^52^ (Table 2). While the medical oncologist typically provides the rationale, goals, and specific requirements for NGS biomarker testing (particularly for iCCA), they may not be part of the patient healthcare team until a diagnosis of iCCA is made. In this scenario, a gastroenterologist or surgical oncologist has the critical role in the diagnostic workflow and must be informed about biomarker testing collection plans.

**Table 2.** Multidisciplinary communication and biomarker testing.

40%-50% of iCCA patients have actionable alterations highlighting the importance of conducting biomarker testing
• 2 FGFR inhibitors received accelerated approval by the FDA for the treatment of patients with previously treated, unresectable locally advanced or metastatic CCA with an *FGFR2* fusion or other rearrangement.^18,19^
• The FDA recently approved the IDH1 inhibitor ivosidenib for adult patients with previously treated, locally advanced or metastatic CCA with an *IDH1* mutation^21,22^
• Other NCCN category 2A options include: NTRK inhibitors (NTRK gene fusion positive tumor), PD-1 inhibitors (MSI-H/dMMR/TMB-H), BRAF/MEK inhibitors (BRAF V600E)
Establish early and clear communication among the Multidisciplinary Team
• Educate the entire multidisciplinary team regarding the importance of biomarker testing
• Optimize the diagnosis of iCCA and emphasize the importance ofcollecting of biopsy samples for biomarker testing at diagnosis
• Consider splitting core samples into separate cassettes to preserve tissue for diagnosis and biomarker testing
• Include ROSE when possible to enhance the quality and quantity of tumor cell content
• Utilize a standardized biopsy requisition form with a scoring system (Supplementary Table S1)
• Implement a feedback loop between the interventional ioncologist, pathologist and medical oncologistmo that focuses on the quality of biopsy samples to ensure the samples meet the biomarker test platform requirements
Include consistent clinical trial meetings molecular tumor boards to help analyze biomarker test results
• Invite interventional oncologist and pathologists to participate in decision making and clinical trial processes

Abbreviations: CCA, cholangiocarcinoma; dMMR, mismatch repair deficient; FGFR, fibroblast growth factor receptor; iCCA, intrahepatic cholangiocarcinoma; IDH1, isocitrate dehydrogenase; MSI-H, microsatellite instability-high; NCCN, National Comprehensive Cancer Network; NTRK, neurotrophic tyrosine receptor kinase; PD-1, programmed cell death protein-1; ROSE, rapid on-site evaluation; TMB-H,tumor mutational burden-high.

To ensure that adequate samples are collected for biomarker testing, the specimen requirements must involve early, formal communication among the multidisciplinary team.^33,39,43,52^ To improve communication, a prescreening biopsy scoring system that identifies lesions suitable for biopsies along with a standardized biopsy requisition form should be considered.^39,52^ A brief description of a prescreening lesion scoring system and sample request form is included in Supplementary Table S1. In fact, the BATTLE-2 and NCI-MATCH trials incorporated a combination of best practices to improve communication and biomarker testing success rates. These practices included utilization of detailed instructions by the interventional oncologist, a prescreening lesion scoring system, inclusion of pathologists, microdissection, and FNA biopsies, along with ROSE.^39^

Adoption of strategies such as inclusion of interventional oncology in the multidisciplinary team and utilization of a biopsy scoring system and standardized biopsy requisition form can improve sample quality and biomarker testing success rates, as well as help identify high-risk patients to minimize complication rates.^50^

### Other Considerations: Timing of Biopsy, Primary Vs Metastatic Lesions, and Liver Transplantation

#### Timing of Biopsy: Stage of Disease

Studies have shown genetic alterations are detectable in patients with surgically resectable iCCA, suggesting early-stage patients may benefit from biomarker testing.^56,57^ However, there is no consensus on when to perform biomarker testing in patients with resectable disease. Experts recommend that the surgical team reserves tissue samples during surgery so biomarker testing can be performed expediently, especially when a preoperative biopsy is not collected. Early tissue collection may expedite biomarker testing and positively influence later treatment planning.^58^ Future studies in early stage iCCA should be further explored so that precision medicine in the perioperative setting can be conducted.

#### Timing of Biopsy: During Treatment Paradigm

While early biomarker testing is recommended to optimize the patient’s treatment plan,^58^ consensus doesn’t exist when considering biomarker testing across lines of therapy. One recent descriptive circulating tumor deoxyribonucleic acid (ctDNA) analysis suggests that chemotherapy may alter the biomarker profile of CCA patients.^59^ Additionally, studies have shown that acquired resistance mutations develop after treatment with FGFR inhibitors.^60,61^ Further research into longitudinal and treatment-induced changes of mutational patterns in iCCA patients is needed.

#### Lesion Choice: Primary Vs Metastatic

The evaluation of tumor heterogeneity and molecular profile differences between primary iCCA and metastatic lesions is up for debate. Recent analysis shows potential biomarker profile differences between primary and metastatic lesions.^61-63^ However, previous analysis didn’t find significant differences in primary and metastatic lesions.^32^ More research is needed to study how the mutational profile differs between primary and metastatic lesions. This analysis will help guide clinicians when considering which lesion to biopsy for biomarker testing.

#### Liver Transplantation

Although liver transplantation (LT) has been contraindicated in iCCA, emerging evidence indicates that select patients with iCCA may be candidates for LT.^64^ Patients who are candidates for LT may not undergo biopsy; therefore, it is critical to apply the inclusion and exclusion criteria of the Mayo Clinic protocol to identify patients for LT.^64,65^

## Expert Discussion and Conclusions

**Figure 1.**
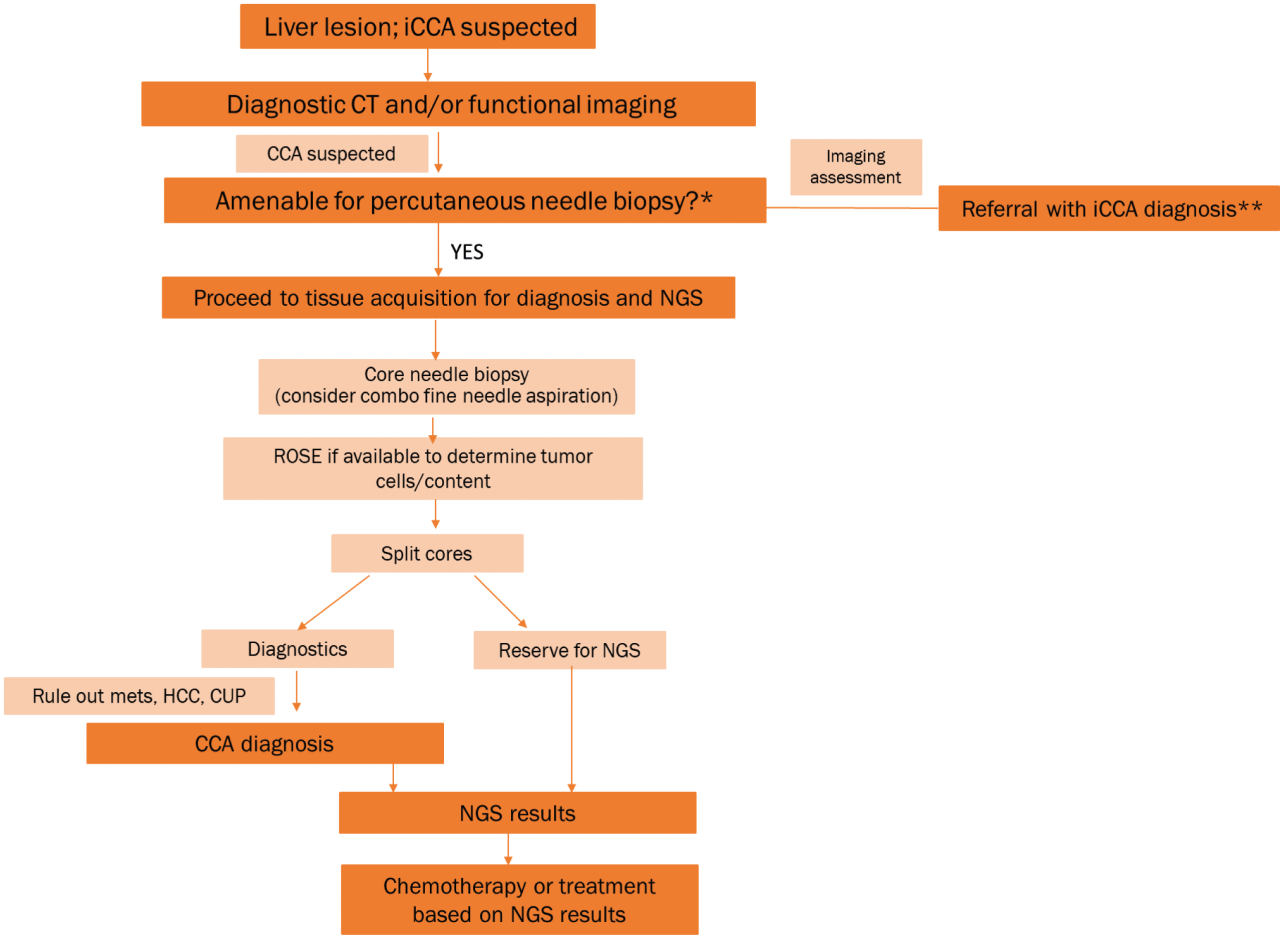
Recommended algorithm for suspected iCCA: Tissue acquisition for diagnosis and biomarker testing. *If no, consider liquid biopsy. **Apply Mayo protocol and if OLT candidate, biopsy should not be performed. If not OLT candidate, continue with process outlined in figure. Abbreviations: CCA, cholangiocarcinoma; iCCA, intrahepatic CCA; CT, computerized tomography; CUP, cancer of unknown primary; HCC, hepatocellular carcinoma; Mets, metastasis; NGS, next-generation sequencing; OLT, orthotopic liver transplantation; ROSE, rapid on-site evaluation.

The clinical relevance of biomarker testing for patients with iCCA is becoming more widely accepted, and the consensus opinion of the multidisciplinary co-authors is that all patients with unresectable or metastatic iCCA should undergo NGS biomarker testing to improve patient outcomes. The panel emphasizes that early communication among the multidisciplinary team is paramount to optimize the biomarker testing process. Integration of cytologic specimens into routine biomarker testing workflows may maximize limited tissue and reduce the need for re-biopsy. A proposed algorithm for diagnostic and biomarker testing is presented in Fig. 1.

Interventional oncology is increasingly being regarded as the fourth pillar of cancer care alongside medical, surgical, and radiation oncology. Therefore, it is crucial that interventional oncologists are included as integral members of multidisciplinary healthcare teams, welcomed at molecular tumor boards, participate as sub-investigators in clinical studies, and help educate the oncology community on the importance of optimizing tissue acquisition for biomarker testing.^66^

Initiatives are needed to “bridge the gap” between academic and community interventional oncologists, especially for rare tumor types such as iCCA. Evidence indicates that collaboration between community and academic oncologists, outreach, and interventions such as telemedicine may allow patients in the community with iCCA to benefit from precision-based medicines.^67^ To establish evidence-based guidelines for iCCA biomarker testing procedures and steer precision medicine decisions, partnerships between national organizations such as the Association for Molecular Pathology (AMP) and the College of American Pathologists (CAP) are needed, similar to currently established partnerships that exist for lung cancer and colorectal carcinoma.^68,69^ Lastly, it is very important that all stakeholders involved in the care of patients with iCCA continue to coordinate their efforts to overcome challenges associated with image-guided biopsies for biomarker testing to improve patient outcomes.

## Supplementary Material

oyac139_suppl_Supplemental_Table_1

## Data Availability

No new data were generated or analyzed in support of this research.
