# Supplementary material for: An Expert, Multidisciplinary Perspective on Best Practices in Biomarker Testing in Intrahepatic Cholangiocarcinoma
Source: Oncologist. 2022 Aug 4;27(10):884–91. doi: 10.1093/oncolo/oyac139 (PMC9526481; doi:10.1093/oncolo/oyac139)
Supplement: oyac139_suppl_Supplemental_Table_1 [file oyac139_suppl_supplemental_table_1.docx]

**Supplemental Table 1. Sample Guide Sheet for Biopsy Collection**

| **Sample Guide Sheet for Biopsy Collection** | |
| --- | --- |
| **Patient Name:** |  |
| **Contact Information** |  |
| Medical Oncologist |  |
| Collaborator - Interventional Oncologist |  |
| Collaborator – Pathologist |  |
| Collaborator – Other |  |
| **ENCOUNTER #1*** | Date: |
| **Purpose of Biopsy** |  |
| Diagnostic |  |
| NGS |  |
| **Biopsy Instructions** |  |
| Lesions that should not be biopsied (eg. previously irradiated, lesions followed for tumor measurements / RECIST) |  |
| Body sites that cannot be biopsied |  |
| **Biopsy collection Requirements** |  |
| Core Needle Size |  |
| Number of Recommended Cores |  |
| Cores for Diagnostic |  |
| Cores for NGS |  |
| FNA (Yes/No) |  |
| First Biopsy Site (by CT/US images) |  |
| Second Biopsy Site (by CT/US images) |  |
| Sample Size |  |
| Minimum Tumor Cell Content Requested |  |
| **MDACC Lesion Score (1/2/3)** |  |
| Biopsy Disposition |  |
| **On-site cytopathologic assessment (available vs not available)** |  |
| **Feedback from the pathologist about sample adequacy** |  |
| Tumor Cell Content |  |
| Cellularity |  |
| Viability of Cells |  |
| NGS Results |  |
| **Signatures** |  |
| **ENCOUNTER #2 (if needed)** | Date: |
| Medical Oncologist |  |
| Interventional Oncologist |  |
| Pathologist |  |
| Other |  |
| **Score 1**: Likelihood of yield: Low; Reason: No target amenable to biopsy, high-risk procedure; Biopsy disposition: Should not be performed.**Score 2**: Likelihood of yield: Medium; Reason: Uncertainty about success either because of technical challenges or lesion characteristics (eg, small size, necrotic, subsolid lesion, sclerotic, not FDG avid, technically difficult biopsy); sclerotic bone lesion usually low yield; Biopsy disposition: Communication with investigator for these types of lesions should occur to determine whether to proceed with biopsy.  **Score 3**: Likelihood of yield: High; Reason: Viable tumor demonstrated on diagnostic imaging (enhancing lesion, growing lesion) that can be sampled aggressively; Biopsy disposition: Proceed with biopsy.  *If ENCOUNTER #2 is needed, the same content as ENCOUNTER #1 may be used | |
| **Reference**: Tam AL, et al. Clin Radiol. 2021;76(2):155.e25-155.e34. | |
